# Effects of veridical expectations on syntax processing in music: Event-related potential evidence

**DOI:** 10.1038/srep19064

**Published:** 2016-01-19

**Authors:** Shuang Guo, Stefan Koelsch

**Affiliations:** 1 Department of Educational Sciences & Psychology, Freie Universität Berlin, Berlin 14195, Germany

## Abstract

Numerous past studies have investigated neurophysiological correlates of music-syntactic processing. However, only little is known about how prior knowledge about an upcoming syntactically irregular event modulates brain correlates of music-syntactic processing. Two versions of a short chord sequence were presented repeatedly to non-musicians (n = 20) and musicians (n = 20). One sequence version ended on a syntactically regular chord, and the other one ended on a syntactically irregular chord. Participants were either informed (cued condition), or not informed (non-cued condition) about whether the sequence would end on the regular or the irregular chord. Results indicate that in the cued condition (compared to the non-cued condition) the peak latency of the early right anterior negativity (ERAN), elicited by irregular chords, was earlier in both non-musicians and musicians. However, the expectations due to the knowledge about the upcoming event (veridical expectations) did not influence the amplitude of the ERAN. These results suggest that veridical expectations modulate only the speed, but not the principle mechanisms, of music-syntactic processing.

When listening for the first time to a piece of music in a given tonality, the underlying harmonic schemata evoke expectancies in the listener regarding a set of possible subsequent events, each with a different probability. How would the processing of a subsequent event be altered if we knew what to expect? According to Meyer, “For if a work has been heard already, we will know what is going to happen and, in later hearings, the improbable will become probable, the unexpected will be expected, and all predictions will be confirmed”^1^. However, it is unknown whether or not expectations about an upcoming event would impact on the processing of such an event in the brain. In the present ERP study, we investigated effects of veridical expectations on the processing of music-syntactic irregularities.

Prediction and expectation are truly at the heart of the perception and cognition of music. We use the term *prediction* as a general term for the overall process of future-directed information processing, and *expectation* as the representation of what is predicted to occur, including a probability distribution, and thus not necessarily representing a single event only (see also ref. 2). Bharucha differentiated between two kinds of expectations^3^: First, *schematic* expectations are based on listeners’ knowledge of regularities about how single musical events are combined into musical sequences (e.g., in Western music, knowledge about combinations of notes, chords and tonalities). Such knowledge about musical schemata (e.g. ref 4) is built up passively, probably over many years. The schematic expectations can either be implicit (e.g., in non-musicians) or explicit (e.g., in musicians). Second, *veridical* expectations refer to listeners’ anticipation of specific events in a familiar event structure. They are generated either by the activation of memory traces for pieces of music, or by explicit prior knowledge of the event structures (i.e., the anticipation of a specific upcoming note, chord etc.). Veridical expectations are usually explicit (e.g., the memory representation of *Happy Birthday*), but can also be implicit (e.g., when playing a piece of music by heart, the motor sequences are often executed automatically, without the player being able to tell explicitly which notes will follow).

The relationship between schematic and veridical expectations is always susceptible to change, depending on the listener’s auditory experience^5^. Justus and Bharucha suggested that these two expectations converge^6^. However, when a listener becomes familiar with irregular/unexpected harmonic progressions of a musical piece, the two expectations diverge. According to Tillmann and Bigand, “veridical and schematic expectations should tap into two different cognitive processes that work independently”^7^. Consonant with this notion, schematic expectations are resistant to veridical expectations: In a study by Justus and Bharucha, schematic expectations for harmonically related chords and veridical expectations for unrelated chords were induced by various situations^6^. It was found that even when unexpected chord pairs occurred more often or when they were preceded immediately by the target pair, schematically expected chords were processed faster (compared to schematically unexpected chord pairs). This indicates that the influence of schematic expectations is stronger than the influence of veridical expectations on chord processing. This is consistent with results of a study by Tillmann and Bigand who tested how far schematic expectations can be influenced by veridical expectations^7^. The results indicate that familiarization with a less-expected musical structure acquired through repeated processing does not reverse response time patterns. This suggests that the influence of schematic knowledge is not weakened by repeated processing of the same structure or by veridical expectations of a specific structure. An ERP study from Pieszek, Widmann, and Schröger^8^ addresses this issue using a cueing oddball paradigm. In this study, a visual cue was presented before each high- or low-pitched tone indicating the pitch of the upcoming tone in most of the trials. Participants were asked to use the cue to discriminate the two sounds as fast as possible. One of high- and low-pitched tones served as auditory standard, the other as auditory deviant. Trials in which the automatic generative model based on auditory-auditory regularity and attended visual-auditory regularity predicted different sounds (contradictory predictions) elicited a Mismatch Negativity (MMN, violation of auditory-auditory regularity) or an Incongruency Response (IR, violation of visual-auditory regularity). Trials in which auditory-auditory regularity and visual-auditory regularity predicted the same sound (redundant predictions) elicited an IRMMN (i.e., a combination of the MMN and the IR). These results provide evidence that auditory sensory predictions based on stimulus-driven extraction of auditory-auditory regularities (similar to schematic expectations in music) and auditory sensory predictions based on knowledge-driven extraction of attended visual-auditory regularities (similar to veridical expectations in music) operate in a highly functionally modular fashion. However, Creel investigated whether listeners activated veridical context memory for a particular melody when processing basic musical properties (harmony and meter, i.e., schematic memory)^9^. It was found that for both harmonic and metrical information, previous familiarization shifted listeners’ preferences towards a probe they had become familiarized with. This suggests that listeners rapidly form specific (veridical) musical memories without explicit instruction.

Musical expectations have been reported to be reflected in a number of neurophysiological markers, such as the P1^10^, the N1^11^, the MMN^12^, the ERAN^13^,^14^ (for a review see ref. 15), and P300-like components^16^,^17^,^18^. Among these components, it was found that the P300 was elicited by the detection of wrong tones occurring in familiar melodies^16^,^18^. In addition to the P300, the ERAN is taken as an electrophysiological reflection of music-syntactic expectancy violations. It is typically maximal at around 150 to 250 ms, and often has a slight right-hemispheric weighting^19^,^20^,^21^,^22^,^23^,^24^,^25^,^26^. ERAN effects have been shown in non-musicians, i.e., in individuals without formal music training and without knowledge of music theory (thus, non-musicians only have implicit knowledge of music-syntactic regularities). In musicians, i.e., in individuals with both implicit and explicit music-syntactic knowledge, the ERAN tends to be larger than in non-musicians (e.g. ref. 27). On the other hand, individuals with dysmusia (or “amusia”) have a smaller ERAN (compared to controls), presumably due to less specific implicit knowledge of musical regularities^28^.

The application of implicit music-syntactic knowledge, as reflected in the ERAN, appears to be automatic or at least partially automatic (e.g. ref. 29), that is, it can be observed even in the absence of voluntarily directed attention^30^,^31^,^32^. Therefore, the present study used the ERAN as an electrophysiological marker of partially automatic processing of music-syntactic irregularities based on implicit long-term knowledge. That is, the ERAN is elicited by irregular, or less regular, music-syntactic events, and hence the violations of schematic expectations evoke ERAN responses (recall that schematic expectations are based on listeners’ knowledge about regularities in the association of notes, chords and tonalities of their cultures in long-term memory).

Previous studies concerned with musical expectations were mainly focused on schematic expectations (behavioral studies: e.g. refs 33, 34, 35; ERPs studies: e.g. refs 13,18,21,36, for a review see ref. 2). Studies addressing veridical expectations used either familiar musical sequences (behavioral studies: e.g. refs 37, 38, 39; ERP studies: e.g. refs 16,18,21), or implicit learning tasks (prior exposure to regular chord sequences, e.g. ref. 20). To our knowledge, only two behavioral studies directly investigated whether schematic expectations are modulated by veridical expectations^6^,^7^. One ERP study by Guo and Koelsch investigated whether veridical expectations (due to the acquisition of veridical knowledge during the course of a supervised learning phase) would modulate the ERAN, the late positive component (LPC) and the P3a^40^. The results indicated that automatic processes related to attention (P3a), but not partly automatic processes of music-syntactic analysis (ERAN) are modulated by veridical expectations. In the present study, we investigated whether or not veridical expectations (due to veridical knowledge) would impact on music-syntactic processing (driven by schematic or style-specific syntactic knowledge) by directly comparing a condition in which predictions can be made with a condition in which no predictions can be made.

To investigate this issue, chord sequences in which the final chord was syntactically either regular or irregular were used. Participants were either informed or not informed about whether the following sequence would end on a regular or an irregular chord. The ERAN was used to test whether partly automatic processes of musical expectation (due to schematic syntactic knowledge of regularities) were modulated by veridical expectations of the regularity of the upcoming final chord (due to the explicit cues): If partly automatic activation of schematic syntactic knowledge (as reflected in the ERAN) were modulated by veridical expectations, the amplitudes of the ERAN would differ between conditions in which participants were informed or not informed about the regularity of the final chord of a sequence. Alternatively, if schematic expectations (based on schematic knowledge) operated independently of veridical expectations (generated by veridical knowledge), then the amplitudes of the ERAN would remain unchanged between these two conditions (with cue and without cue about the final chord). We obtained data from two groups of subjects, musicians and non-musicians. Data from musicians were acquired because they have more specific knowledge of musical regularities than non-musicians (see above), and it was of interest to test whether such expertise would impact on the processing of expectations based on veridical vs. schematic knowledge (compared to non-musicians).

## Results

### Behavioral results

The mean percentages of correct responses of regularity judgments in the pre- and post-tests were 63.5% (*SD* = 16.63%) and 70.5% (*SD* = 16.93%) in non-musicians. Musicians achieved 97% (*SD* = 5.94%, pre-test) and 99.25% (*SD* = 2.45%, post-test) correct responses. Correct responses of regularity judgments were above chance in the pre-test for both non-musicians and musicians (*p* < 0.01 in both one-sample *t*-tests). An ANOVA with factors pre-/post-test and group indicated an effect of pre-/post-test (F(1,38) = 4.31, *p* < 0.05, partial η^2^ = 0.1), an effect of group (F(1,38) = 95.47, *p* < 0.001, partial η^2^ = 0.72), but no interaction between factors pre-/post-test and group (*p* = 0.29).

### ERP results

#### ERAN

**Figure 1:**
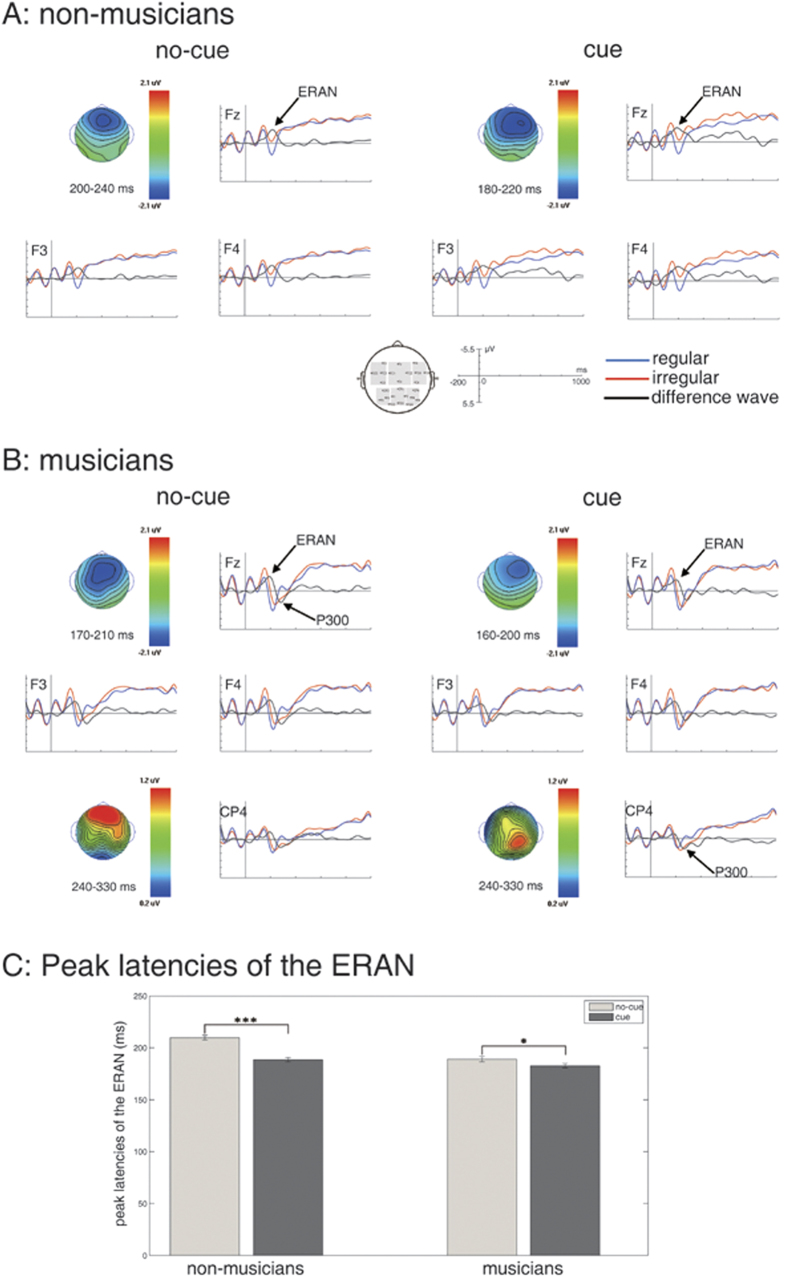
The upper panel (A) shows grand-average of ERPs elicited by regular (blue) and irregular (red) chords in the conditions in which participants were not cued (non-cued condition) or cued (cued condition) as to whether the final chord was regular or irregular for non-musicians (n = 20). The black waveform shows difference potentials (regular subtracted from irregular chords). Compared to regular chords, irregular chords elicited an early right anterior negativity (ERAN, indicated by arrows). Isopotential maps of the ERAN (difference potentials, regular subtracted from irregular chords) in the non-cued and the cued conditions are shown. The inset shows the ROIs used for statistical analyses (shaded in gray). The middle panel (**B**) shows ERPs elicited by regular (blue) and irregular (red) chords in the non-cued and the cued conditions in musicians (n = 20). Compared to regular chords, irregular chords elicited an ERAN and a P300 (indicated by arrows). The P300 had a more frontal preponderance in the non-cued condition, and a more posterior preponderance in the cued condition. Isopotential maps of the ERAN and the P300 (difference potentials, regular subtracted from irregular chords) in the non-cued and the cued conditions are shown. The bottom panel (**C**) shows peak latencies of the ERAN (difference potentials, regular subtracted from irregular chords) in the non-cued and the cued conditions in non-musicians and musicians. The data show that the latency differences between the non-cued and the cued conditions were statistically significant for both non-musicians and musicians. Error bars indicate standard error of mean (SEM). ^*^p < 0.05, ^**^p < 0.01, ^***^p < 0.001.

Figure 1 shows the grand-average ERPs in the non-cued and the cued conditions, separately for non-musicians (Fig. 1A) and musicians (Fig. 1B). In both non-musicians and musicians, the irregular chords (compared to regular chords) elicited an ERAN that was maximal at anterior scalp sites in both the non-cued and the cued conditions. The peak latency of the ERAN was earlier in musicians than in non-musicians, and was in both groups earlier in the cued condition than in the non-cued condition: The ERAN of non-musicians/musicians had a peak latency of 210 ms (SD = 11 ms)/189 ms (SD = 11 ms) in the non-cued condition (where participants were not informed as to whether the final chord would be regular or irregular), and 189 ms (SD = 9 ms)/183 ms (SD = 9 ms) in the cued condition (where participants were informed as to whether the final chord would be regular or irregular). An ANOVA analyzing the ERAN peak latencies with factors cue and group revealed an effect of cue (F(1,38) = 46.82, *p* < 0.001, partial η^2^ = 0.55), an effect of group (F(1,38) = 27.34, *p* < 0.001, partial η^2^ = 0.42), and an interaction between cue and group (F(1,38) = 13.90, *p* = 0.001, partial η^2^ = 0.27). Paired-sample *t*-tests showed that the latency difference between the non-cued and the cued conditions was statistically significant for both non-musicians (*t* = −6.35, *p* < 0.001, Cohen’s *d* = 2.12) and musicians (*t* = −2.81, *p* < 0.05, Cohen’s *d* = 0.60; see Fig. 1C).

To investigate whether the peak latencies of the ERAN in the non-cued and the cued conditions have a behavioral correlate, we computed correlations between the change of peak latencies of the ERAN (peak latency of the ERAN in the cued condition subtracted from peak latency of the ERAN in the non-cued condition) and the change of regularity judgments (correct responses of regularity judgments in the pre-test subtracted from correct responses of regularity judgments in the post-test) across all participants. Correlation analysis showed that there was a marginally significant correlation between the change of peak latencies of the ERAN and the change of regularity judgments (*r* (38) = 0.26, *p* = 0.05) across all participants.

To analyze the ERAN amplitudes statistically, different time windows for non-musicians and musicians were used in a global ANOVA that was computed for the time windows from 190 to 230 ms (non-cued condition, non-musicians), from 170 to 210 ms (cued condition, non-musicians), from 170 to 210 ms (non-cued condition, musicians), and from 160 to 200 ms (cued condition, musicians) with factors regularity, cue, anterior-posterior distribution, hemisphere and group. This ANOVA indicated an effect of regularity (F(1,38) = 62.86, *p* < 0.001, partial η^2^ = 0.62), an effect of cue (F(1,38) = 5.84, *p* < 0.05, partial η^2^ = 0.13), but no interaction between regularity and cue (*p* = 0.87; see Table 1 for all significant results of this ANOVA). Due to an interaction between regularity, anterior-posterior distribution and hemisphere (F(2,76) = 6.84, *p* < 0.01, partial η^2^ = 0.15), paired-sample *t*-tests were computed to compare ERPs elicited by regular and irregular chords, separately for the right (*t* = 7.94, *p* < 0.001, Cohen’s *d* = 0.96), central (*t* = 8.05, *p* < 0.001, Cohen’s *d* = 0.94) and left (*t* = 8.02, *p* < 0.001, Cohen’s *d* = 0.90) anterior ROIs (*p* > 0.2 for the right, central and left posterior ROIs), reflecting that the ERAN was significant over all anterior ROIs, with the largest amplitude over central anterior leads (see also amplitude values of right, central and left anterior ROIs in Table 2). Even when computing an ANOVA for the central anterior ROI (calculated despite the missing interaction involving cue and regularity in the global ANOVA), no interaction between regularity and cue was indicated (*p* = 0.95). This result provides assurance that the ERAN effects did not differ between cued and non-cued conditions (and that the missing interaction between regularity and cue in the global ANOVA was not simply a statistical artifact).

#### P300

The ERAN was followed by a P300 in musicians (see Fig. 1B). The peak latency of the P300 was around 285 ms. A mixed-model ANOVA for the time window from 240 to 330 ms with factors regularity, cue, anterior-posterior distribution, hemisphere and group was computed (see Table 1 for detailed results). The results indicated an interaction between regularity and group (F(1,38) = 10.31, *p* < 0.01, partial η^2^ = 0.21), but no interaction involving regularity and cue (*p* = 0.8). The interaction between regularity and group was due to a difference between non-musicians and musicians in the P300 amplitude evoked by irregular chords compared to regular chords (see also Fig. 1): Paired-sample *t*-tests comparing ERPs between regular and irregular chords, conducted separately for non-musicians and musicians, showed that the P300 was elicited by irregular chords in musicians (*t* = −3.55, *p* < 0.005, Cohen’s *d* = 0.68; see also Fig. 1B), but not in non-musicians (*p* = 0.29).

#### Penultimate chord

Results of the penultimate chord are provided in the Supplementary Information (see Supplementary SI Text, Figure S1, Table S1).

## Discussion

### Behavioral results

All participants (non-musicians and musicians) were able to differentiate above chance between regular and irregular chords in the pre-test. The ability to differentiate between regular and irregular sequences in the pre-test was based on implicit knowledge in non-musicians and probably both implicit and explicit knowledge in musicians (this issue was not addressed in this study). Correct responses of regularity judgments increased from pre-test to post-test across all participants, providing assurance that participants paid attention to the cues in the experiment. Correct responses differed significantly between non-musicians and musicians in the pre-test. This is most probably due to the fact that musicians are more sensitive and accurate at processing syntactic irregularities than non-musicians. The performance of regularity judgments in musicians was nearly 100% in the post-test, suggesting that musicians did not have any problems differentiating regular from irregular chords. It is, therefore, highly likely that musicians could well predict the regular and irregular final chords when they were cued. That is, they could generate veridical expectations in the cued condition (this was also confirmed by the musicians during debriefing).

### ERP results

The present study aimed at investigating whether music-syntactic processing (based on schematic syntactic knowledge) would be modulated by veridical expectations (due to veridical knowledge). Our experiment was set up to test whether and how the ERAN and P300 would be modulated by veridical expectations.

In the most extreme case, one might argue that once participants knew what was going to happen, the ERAN-response might not be evoked at all. Strictly speaking, after having veridical expectations for the irregular final chord (due to the cue), this chord did not violate any “expectation for a regular chord” anymore. However, our experiment suggests that irregular chords did elicit a clear ERAN in the cued condition, even in musicians who could predict the regular and irregular final chords. It seems likely that in the cued condition, veridical expectations for the irregular sequence ending were confirmed, but the schematic expectations for the regular ending were violated. This notion is supported by a study by Miranda and Ullman^21^ which suggests that schematic expectations (generated by the activation of implicit stylistic knowledge) and veridical expectations (generated by the activation of memory traces for specific musical pieces) have distinct neural correlates. In that study^21^, out-of-key deviant notes that violated tonal harmony rules in unfamiliar melodies elicited the ERAN, and in-key deviant notes in familiar melodies elicited an N400. This is consistent with the notion proposed by Dowling and Harwood^41^, as well as by Meyer^1^ that schematic expectations may work at an automatic, or “subconscious” level^7^ (p. 220). The absence of an N400 in our study is probably due to the fact that the veridical expectations were not violated but only generated (cued condition) or not (non-cued condition).

Several studies investigated effects of predictive processes on auditory sensory memory operations using the MMN^42^,^43^,^44^,^45^,^46^,^47^. These studies suggest that the MMN is not affected by predictive information. For example, Sussman, Winkler, and Schröger^43^ presented a visual cue before each auditory stimulus, and found that the MMN was not affected by the predictability of a deviant tone. Similarly, using a cueing oddball paradigm, Ritter, Sussman, Deacon, Cowan, and Vaughan^44^ showed that the MMN was elicited even when the deviant tone was congruently cued by a visual stimulus (thus consciously expected by the participants). We presume that the ERAN elicited in our study is so strongly driven by the schematic syntactic knowledge stored in long-term memory, that it cannot simply be overridden by veridical expectations. This parallels the fact that the MMN is so strongly driven by acoustical (low-level) predictions that it is resistant to veridical knowledge of an upcoming event.

The peak latency of the ERAN was earlier in the cued condition (189 ms in non-musicians and 183 ms in musicians) compared to the non-cued condition (210 ms in non-musicians and 189 ms in musicians). As Schmuckler^48^ suggested, knowing about the occurrence of a particular event raises one’s expectation for that event. The shorter latency of the ERAN in the cued condition (for non-musicians and musicians) is likely due to more specific expectations for the final chord, which facilitated the processing of the final chord in the cued condition than in the non-cued condition. This is in line with studies showing a response delay for unpredictable auditory stimuli compared to predictable ones^43^,^44^. The reduction of the peak latency of the ERAN in the cued condition compared to the non-cued condition (as reflected in the peak latency difference of the ERAN in the non-cued and the cued conditions) has a behavioral correlate (as reflected in the correct responses difference of regularity judgments in the pre- and post-tests). This indicates that the speed of music-syntactic processing is related to the ability of acquiring veridical knowledge about the irregularities across all participants.

Our results show that schematic knowledge cannot be easily overridden by veridical expectations, thus resulting in the ERAN amplitude not being affected; however, veridical expectations may lead to a facilitated processing of the irregular event, reflected in the reduced ERAN latency. Previous studies showed that the degree of music-syntactic irregularity affects the amplitude, but much less the latency of the ERAN (e.g. refs 13,19). This suggests that the amplitude of the ERAN reflects the music-syntactic processing steps (i.e., the neural computations performed). In line with the notion, veridical expectations did not influence the processing steps of musical regularities in our study. Regarding the peak latency of the ERAN, previous studies using harmonic sequences similar to those used in the present study reported ERAN latencies, between 170 and 220 ms. However, studies reporting longer ERAN peak latencies (e.g. refs 49,50) used non-repetitive sequences, in which the position of irregular chords was unpredictable. In our study, the final chords were unpredictable in the non-cued condition, which possibly led to a longer latency of the ERAN than in the predictable cued condition. That is, we assume that the processing of musical regularities itself was facilitated by predictions. For example, the phenomenon of an unaffected ERAN amplitude and a reduced ERAN latency in our study is reminiscent of the phenomenon that carrying out a complex action sequence becomes faster once it has been carried out several times, although the amount of processing steps remains the same.

A P300 was elicited by irregular chords in musicians, but not in non-musicians. In the non-cued condition, the P300 had a more frontal distribution (P3a), and in the cued condition, the P300 had a more parietal distribution. The frontal P300/P3a elicited in the non-cued condition probably reflects allocation of attentional resources in response to the irregular chords^51^. The P300 elicited in the cued condition tended to have a more parietal distribution. One possibility is that this effect reflects a shift in the scalp distribution of the P3a as an effect of predictive processes. This notion is consistent with results of a study by Horváth, Sussman, Winkler, and Schröger^52^ who suggested that the scalp distribution of the P3a is more posterior when participants could predict an auditory “oddball” (a visual stimulus signaled whether the forthcoming sound was a deviant or a standard), compared to when the auditory deviant was unpredictable. However, it is also possible that, when predictable, attention-switching processes elicited by irregular chords were reduced (thus leading to smaller, or no P3a potentials), and that P3b potentials were elicited reflecting, e.g., the confirmation of the expectation for an irregular event. This notion is supported by findings of language processing studies on the processing of target words in highly constraining sentence contexts (such as, antonyms^53^, idioms^54^, or collocations^55^). The P3b is elicited only by highly expected words (e.g., The opposite of *black* is *white*) compared to less expected endings (e.g., The opposite of *black* is *yellow*).

It is also of interest that the ERAN was shown in both musicians and non-musicians, whereas P300 potentials were observed only in musicians. This suggests that the neuro-cognitive mechanisms reflected in P300 potentials (supposably related to attention allocation or confirmation of expectations) are influenced more strongly by musical expertise than the processes of music-syntactic analysis (as reflected in the ERAN).

If schematic expectations do operate independently of veridical expectations, regardless of what information listeners have about the sequences, future studies could investigate whether the fulfillment and violation of schematic expectations would be modulated by, or interact with the validity of the veridical expectations generated by competing information of what to come (e.g., providing participants with wrong cues of the sequences using a cue-validity method). Because veridical knowledge provided in this study is not enough for participants to form very specific expectations, another study could be conducted to determine the effects of very specific veridical knowledge on the processes underlying the generation of the ERAN, by measuring subjects repeatedly across the same EEG-method taking place on several days (i.e., over longer periods of exposure). Moreover, because previous studies regarding the strength of schematic expectations were conducted in the field of music perception, future studies may explore what roles schematic and veridical expectations play in music production (e.g., when participants play musical pieces themselves). Furthermore, in auditory prediction studies, prediction effects on the auditory ERP can be strongly modulated by others factors like attention, or context (for a review see refs 56,57). In our study, we did no assess how much attention participants paid to the chord sequences. Therefore, future studies could control attention by giving a specific task to participants (e.g. rate how surprised participants feel about the ending of the presented sequence) to ensure that they paid the same amount of attention to each of the stimuli. Thus, conclusions drawn from the present experiment are only valid within the current experimental context.

In conclusion, the results of present study indicate that the processing of violations of schematic expectations (as reflected in the ERAN) is not abolished by veridical expectations. However, veridical expectations modulate the speed of music-syntactic processing. On a more general level, our findings indicate that veridical expectations of a musical work do not disrupt the formation of more general schematic expectations. This helps to clarify the role of veridical expectation and its contribution to the modulation of music-syntactic processes. The results offer a solution to Meyer’s query in “On rehearing music”^1^, that is, no matter whether previous (veridical) knowledge exists or not, schematic knowledge (on which predictions are based) functions independently. In the context of predictive coding and free energy minimization^58^, this means that veridical knowledge affording the opportunity to resolve uncertainty in the future is dependent of, and can even conflict with, implicit knowledge. Thus, being aware of the present results, we may know that we will make a wrong prediction when listening to a deceptive cadence in a known piece of music.

## Methods

### Participants

The current study was approved by the ethics committee of the Psychology department of the Freie Universität Berlin and has been performed in accordance with ethical standards outlined by the Declaration of Helsinki. Written informed consent was obtained from all participants. Twenty non-musicians and 20 musicians participated in the experiment (age-range: 19–34 years, *M* = 25.15 years, 10 males and 10 females in each group). Non-musicians had not received any formal musical training besides normal school education. Musicians had studied either musicology or an instrument for at least 2 years at a conservatory (range: 2–7 years, *M* = 4.5 years), and had all received at least 10 years of instrumental training (i.e., piano, violin, viola, cello, accordion, fife, singing). All participants had normal hearing (according to standard pure tone audiometry) and no neurological or psychiatric disorder (according to self-report).

### Stimuli and apparatus

**Figure 2:**
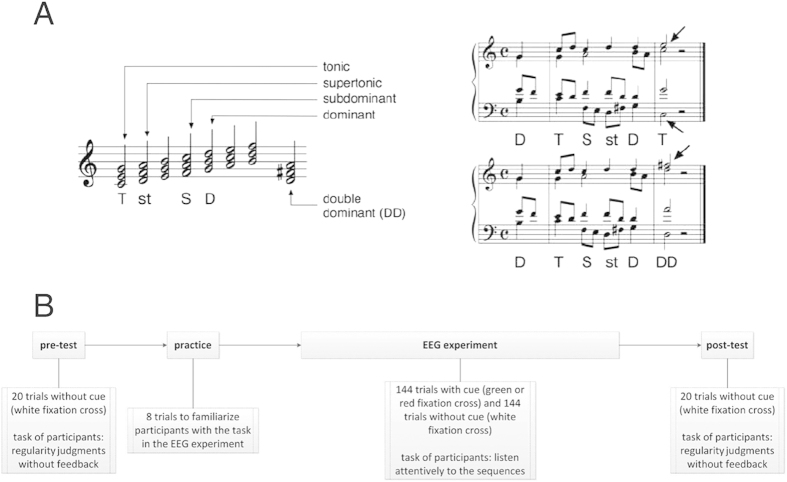
Example of experimental stimuli and experimental procedure. The left panel of (**A**) illustrates the chord functions. The chord built on the first scale tone is denoted as the tonic (T), the chord on the second scale tone as the supertonic (st), on the fourth scale tone as subdominant (S), and on the fifth scale tone as the dominant (D). The major chord on the second tone of a scale can be interpreted as the dominant to the dominant, or double dominant (DD). The right panel of (**A**) illustrates examples of the polyphonic sequences in C major (duration of each sequence was 3 s). Chord sequences ended either on a tonic chord (T, regular), or on a double dominant (DD, irregular). Figure 2(A) was obtained from ref 14. Arrows indicate pitches that were not contained in the preceding chords. The lower panel (**B**) illustrates the experimental procedure: It consisted of pre-test, practice, EEG experiment, and post-test. During the EEG experiment (but not during pre- or post-test), participants were either informed (by a green or a red fixation cross, cued condition), or not informed (by a white fixation cross, non-cued condition) about whether the sequence would end on the regular (tonic) or the irregular (DD) chord, so that participants could learn to predict regular and irregular endings in the cued condition.

The stimuli were polyphonic (four-part) sequences that had been used in previous studies (e.g. ref 15). There were two versions of sequences, both sequence versions were identical except the final chord, which was regular (tonic) in one sequence version, and syntactically irregular (dominant to the dominant, DD) in the other (for an illustration see Fig. 2A). Sequences began with a dominant upbeat, followed by a tonic, a subdominant, a supertonic, and a dominant. Additionally, eighth notes (auxiliary and passing notes) were introduced in a polyphonic fashion. Presentation time of each chord was 500 ms, except for the final chord which lasted 1000 ms. Sequences were transposed to twelve major keys, resulting in 24 (2 × 12) different sequences. Note that the first chord of each sequence (dominant upbeat) only implied the tonic of the sequence, thus regular and irregular final chords could not simply be detected by comparing them to the first chord of each sequence. Moreover, sequences were composed in a way in which syntactically irregular chords (DDs) introduced only one new pitch, whereas regular chords (tonic) introduced two new pitches (see arrows in Fig. 2A). Therefore, any mismatch response evoked by irregular chords (DDs) compared to regular chords (tonic) could not simply be due to the processing of deviant pitches.

### Procedure

Participants listened to the stimuli through headphones (60 dB SPL). The procedure consisted of pre-test, practice, EEG experiment, and post-test (see Fig. 2B for an illustration). A *pre-test* was conducted with 20 trials. The purpose of this pre-test was to assess participants’ capability to differentiate between regular and irregular sequence endings. In each trial, a sequence was presented with equiprobably regular or irregular ending, after which participants indicated whether the final chord of the sequence sounded regular or irregular by pressing one of two response buttons. There was no feedback on their judgments. Trials were self-paced (i.e., participants determined when to proceed with the next trial by pressing a button). Duration of the pre-test was around 3 min. Subsequently, eight practice trials were delivered to familiarize participants with the task in the EEG session. After the practice trials, EEG experiment started. In each of the trials, participants were either informed or not informed about whether the following sequence would end on a regular or an irregular chord: Beginning with the presentation of a sequence, a green fixation cross indicated that the sequence was a regular version, while a red fixation cross indicated that the sequence was an irregular version, and a white fixation cross indicated that the sequence was either a regular version or an irregular version (i.e., without telling participants what the final chord of the sequence was going to be). The task of participants was to listen attentively to the sequences. They were not asked to learn, or detect, regular or irregular endings, to avoid that the ERAN elicited by irregular chords would be overlapped with N2b and P3 potentials. Thus, over the course of the experiment, participants learned to predict the cued final chord, but were not able to predict the non-cued final chord. Regular and irregular sequences occurred equiprobably (*p* = 0.5) and were pseudorandomly intermixed in a way that no more than three sequences of the same version (regular, irregular) followed each other, and that each sequence was presented in a tonal key that differed from the key of the preceding sequence. In the EEG experiment, each sequence was presented 12 times, amounting to 288 (2 × 12 × 12) trials in total. Thus, there were 144 cued trials and 144 non-cued trials. The experiment consisted of four blocks, each with a duration of 7 min. After the EEG experiment, a *post-test* was conducted which was identical to the pre-test. The duration of the entire EEG session (with pauses between blocks) amounted to approximately 35 min.

The comparison between performance of pre- and post-tests allowed us to ensure that participants paid attention to the cues of the EEG experiment, because only by attending to the cues in the EEG experiment could participants have increased their performance in the post-test (compared to the pre-test).

We did not use non-symbolic methods chosen in previous behavioral studies to provide veridical information (e.g., providing a preview of chords, or a repetition priming paradigm)^7^,^8^, because only by informing participants of whether the following sequence was regular or irregular with a cue (i.e., by using symbolic information), they could acquire the veridical knowledge of syntactic regularities. We did not use a repetition priming paradigm due to the length of our experimental session.

### EEG data recording

The electroencephalogram (EEG) was recorded with a BrainAmp MR plus amplifiers system (Brain Product Inc., Gilching/Germany) from 59 electrodes (Fp1, Fpz, Fp2, AF7, AF3, AF4, AF8, F7, F5, F3, F1, Fz, F2, F4, F6, F8, FT7, FC5, FC3, FC1, FC2, FC4, FC6, FT8, T7, C5, C3, C1, Cz, C2, C4, C6, T8, TP7, CP5, CP3, CP1, CPz, CP2, CP4, CP6, TP8, P7, P5, P3, P1, Pz, P2, P4, P6, P8, PO7, PO3, POz, PO4, PO8, O1, Oz, O2; extended international 10–20 system), referenced to the left mastoid (M1). Four electrodes were used for recording the vertical and the horizontal electrooculogram (EOGs). The ground electrode was located on the sternum. The EEG was digitized at a rate of 500 Hz (low and high cut off were D.C. and 1000 Hz, respectively) and the impedances were kept below 5 kΩ.

### EEG data analysis

Data were analyzed offline using EEGLAB v9.0.4.4b^59^. Raw data were filtered with a 49–51 Hz band-stop filter with finite impulse response (FIR, filter order: 2750 points) to eliminate line noise, and a 0.25 Hz high-pass filter (FIR, filter order: 13750 points) to remove slow waves (such as electrode saturation or drifts). An independent component analysis (ICA) was carried out, and components representing artifacts (eye blinks, eye movements, and muscle activity) were removed. Afterwards, data were filtered with a 25 Hz low-pass filter (FIR, filter order: 550 points) to remove remaining high-frequency noise (such as muscle activity that was not removed using the ICA). Subsequently, data were epoched relative to the onset of the final (regular or irregular) chord, and rejected when epochs contained any of the remaining artifacts: (a) if amplitudes exceeded ± 100 μV or if linear trends exceeded 100 μV in a 300 ms gliding time window, (b) if an epoch was lying outside a ± 5 SD range (for a single channel) or a ± 3 SD range (for all channels) of the mean probability distribution, (c) if the data were lying outside a ± 5 SD range (for a single channel) or a ± 3 SD range (for all channels) of the mean distribution of kurtosis values (thus excluding abnormally distributed data), (d) if spectra deviated from the baseline spectrum by ± 30 dB in a 0 to 2 Hz frequency window (thus excluding data with improbable spectra), and (e) by visual inspection (to eliminate small blinks and drifts that were not rejected by the automatic procedures). Then, data were re-referenced to the algebraical mean of left and right mastoid leads. Finally, non-rejected epochs were averaged from −200 to 1200 ms relative to the onset of the final (regular or irregular) chord with a −200 to 0 ms baseline. Each condition (regular without cue, irregular without cue, regular with cue, and irregular with cue) had a maximum of 72 trials. Each participant had 64 artifact-free trials on average and 60 artifact-free trials at least per condition.

For statistical analyses, mean amplitude values of the ERAN and P300 were computed for six regions of interest (ROIs): left anterior (F3, C3, FC5, FC3), central anterior (Fz, Cz, FC1, FC2), right anterior (F4, C4, FC6, FC4), left posterior (P3, CP3, PO3, P5), central posterior (P1, P2, CPz, POz), and right posterior (P4, CP4, PO4, P6). Data of all electrodes were used to compute isopotential maps.

Repeated measures ANOVAs were conducted for the amplitudes of the ERAN and the P300 with the within-subjects factors regularity (regular, irregular), cue (without cue, with cue), anterior-posterior distribution (anterior, posterior ROIs), and hemisphere (left, central, and right ROIs). Within these ANOVAs, the between-subjects factor group (non-musicians, musicians) was included. Main effects and interactions of the factor hemisphere were adjusted using the Greenhouse-Geisser correction. Cohen’s *d* (for *t*-tests) and partial η^2^ (for ANOVAs) were used as measures of effect sizes. Cohen’s *d* was calculated using the formula provided in^60^ (d-values of 0.2 correspond to small effect-size, 0.5 to medium effect-size, and 0.8 to large effect-size). Partial η^2^ was calculated in SPSS (partial η^2^ values of 0.01 are defined as small effect, 0.06 as medium effect, and 0.138 as large effect)^60^.

Peak latencies of the ERAN were determined for each subject using the difference-waveforms of ERPs (regular subtracted from irregular chords). The search windows in the non-cued and cued conditions were determined using the grand-average difference-waveforms of ERPs: It started with the earliest statistically significant sampling point taken from musicians in the cued condition (160 ms), and ended with the latest statistically significant sampling point taken from non-musicians in the non-cued condition (230 ms). For each subject, the most negative peak value (peak amplitude) was in this search window (160 to 230 ms), and it was checked manually that the actual ERAN-peak of each participant’s data fell into this search window.

Time windows for statistical analyses of the ERAN and the P300 amplitudes were centered around the peak amplitudes. Latency of peak amplitudes was determined for the P300 using the grand-average difference-waveforms of ERPs. Because the peak latency of the ERAN differed between groups, different time windows were chosen for non-musicians and musicians. For non-musicians, the time windows were 190 to 230 ms (ERAN in the non-cued condition) and 170 to 210 ms (ERAN in the cued condition). The time windows for musicians were 170 to 210 ms (ERAN in the non-cued condition) and 160 to 200 ms (ERAN in the cued condition). The time window of the P300 for all participants was 240 to 330 ms. For presentation purposes, averaged data were filtered after statistical evaluation (10 Hz low-pass, 41 points, FIR).

## Additional Information

**How to cite this article**: Guo, S. and Koelsch, S. Effects of veridical expectations on syntax processing in music: Event-related potential evidence. *Sci. Rep.* **6**, 19064; doi: 10.1038/srep19064 (2016).

## Supplementary Material

Supplementary Information

## Figures and Tables

**Table 1:** Summary of global ANOVAs for the amplitudes of the ERAN and the P300 with factors regularity (regular, irregular), cue (without cue, with cue), anterior-posterior distribution (anterior, posterior ROIs), hemisphere (left, central, and right ROIs) and group (non-musicians, musicians).

**Factors**	**ERAN**		**P300**	
	***df***	***F***	***df***	***F***
Regularity	1,38	62.86***		
Cue	1,38	5.84*		
Group			1,38	30.55***
Hem.	2,76	8.64**	2,76	4.06*
Regularity × AntPost	1,38	26.07***		
Regularity × Hem.	2,76	6.21**		
Regularity × Group			1,38	10.31**
Cue × Hem.	2,76	4.57*		
Group × AntPost	1,38	7.69**	1,38	4.68*
Group × Hem.	2,76	4.08*	2,76	10.67***
AntPost × Hem.	2,76	4.70*		
Regularity × AntPost × Hem.	2,76	6.84**		

Only significant results (main effects and interactions) with *p* < 0.05 are listed. Significance of *p* values is indicated by asterisks (^*^p < 0.05, ^**^p < 0.01, ^***^p < 0.001).

**Table 2:** Summary of the ERAN amplitudes (difference potentials: regular subtracted from irregular chords) in the non-cued and the cued conditions for all participants (mean, with standard deviation in parentheses).

**condition**	**all participants (n** **=** **40)**		
	**right anterior ROI**	**central anterior ROI**	**left anterior ROI**
no-cue (μV)	−1.50 (1.71)	−1.76 (2.02)	−1.38 (1.68)
cue (μV)	−1.66 (1.63)	−1.73 (1.79)	−1.36 (1.43)
