# Supplementary material for: Effects of veridical expectations on syntax processing in music: Event-related potential evidence
Source: Sci Rep. 2016 Jan 19;6:19064. doi: 10.1038/srep19064 (PMC4726113; doi:10.1038/srep19064)
Supplement: Supplementary Information [file srep19064-s1.pdf]

Effects of veridical expectations on syntax processing in music: Event-related potential evidence

Shuang Guo*, Stefan Koelsch

Supplementary Information

Figure

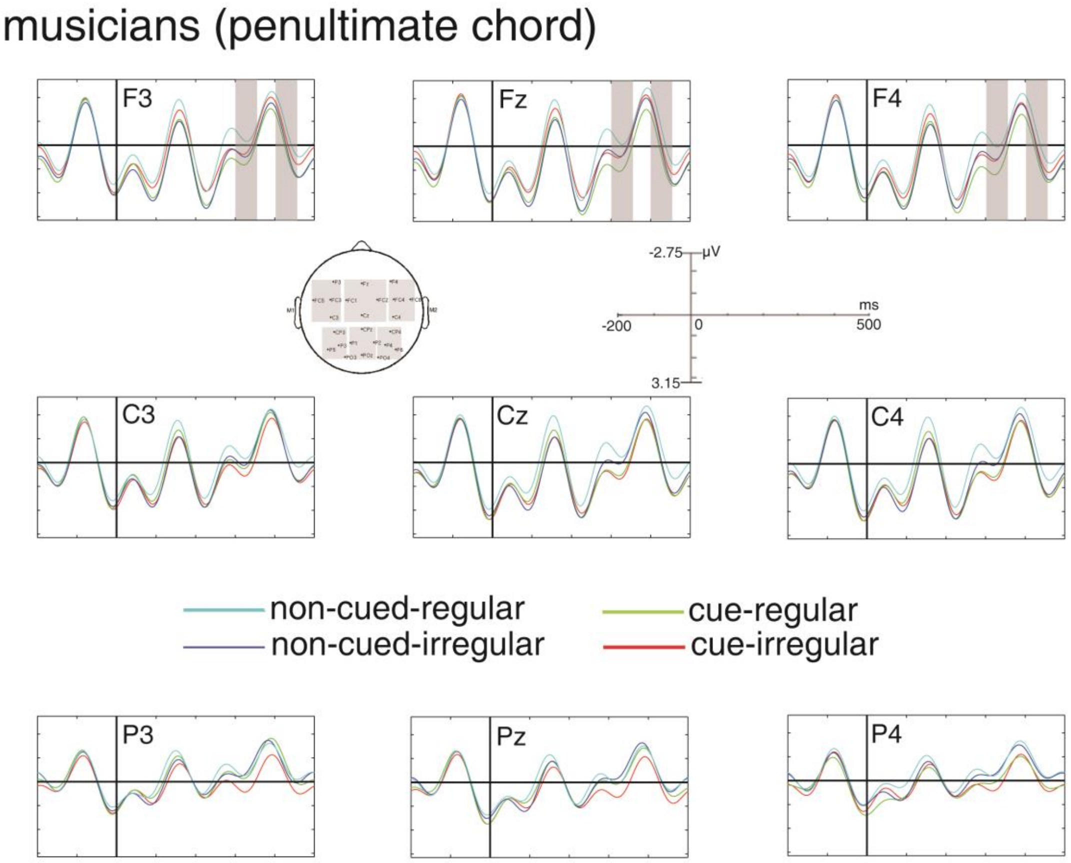

Figure S1. Grand-average of ERPs elicited by the penultimate chord (the dominant chord before the final tonic chord) in musicians. The cyan waveform shows ERPs evoked by regular chords in the non-cued condition, the blue waveform shows ERPs evoked by irregular chords in the non-cued condition, the green waveform shows ERPs evoked by regular chords in the cued condition, and the red waveform shows ERPs evoked by irregular chords in the cued condition. The ERPs elicited in the cued condition were larger than in the non-cued condition over anterior leads (indicated by gray-shaded areas). The inset shows the ROIs used for statistical analyses (shaded in light gray).

Table

Table S1. Summary of global ANOVAs for penultimate chord with factors regularity (regular, irregular), cue (without cue, with cue), anterior-posterior distribution (anterior, posterior), hemisphere (left, central, and right) and group (non-musicians, musicians). Only time windows with significant results (main effects and interactions) are listed. Significance of *p* values is indicated by asterisks ($^{*}p < 0.05$, $^{**}p < 0.01$, $^{***}p < 0.001$).

Factors	50-100 ms		250-300 ms		300-350 ms		400-450 ms	
	df	*F*	*df*	*F*	*df*	*F*	*df*	*F*
Regularity	1,38	4.18*	1,38	3.94*				
Cue					1,38	5.05*		
Cue × AntPost					1,38	3.89*		
Cue × Hem.							2,76	3.41*
Regularity × Cue × AntPost			1,38	4.71*				
Regularity × Cue × AntPost × Group					1,38	3.76*	1,38	7.79**
Regularity × Cue × AntPost × Hem. × Group			2,76	2.91*				

SI Text

Penultimate chord. To investigate possible ERP correlates of predictive processes preceding the final chord, we also investigated ERPs elicited by the penultimate chord (i.e., the dominant chord preceding the final tonic chord).

FIGURE S1 AROUND HERE

Because we did not have prior hypotheses, ANOVAs were computed for 50 ms windows (0-50 ms, 50-100 ms, 100-150 ms, 150-200 ms, 200-250 ms, 250-300 ms, 300-350 ms, 350-400 ms, 400-450 ms, 450-500 ms) of the penultimate chord with factors regularity, cue, anterior-posterior distribution, hemisphere and group (see Table S1 for detailed results).

TABLE S1 AROUND HERE

For the time window from 250 to 300 ms, the analysis indicated an interaction between regularity, cue, anterior-posterior distribution, hemisphere and group (and visual inspection indicated that this interaction was due to a difference between ERPs in the cued compared to the non-cued condition in musicians at right anterior electrodes; see also Fig. S1). Therefore, follow-up ANOVAs were conducted, separately for each of the anterior ROIs with factors regularity, cue and group, indicating an effect of group ($F(1,38) = 4.54$, $p < 0.05$, partial $\eta^2 = 0.11$) over right anterior leads. An independent-samples *t*-test comparing ERPs between non-musicians and musicians showed that ERPs differed between groups over right anterior leads ($t = -2.13$, $p < 0.05$, Cohen's $d = 0.67$).

For the time windows from 300 to 350 ms and 400 to 450 ms, the analyses indicated an interaction between regularity, cue, anterior-posterior distribution and group. Follow-up ANOVAs conducted separately for non-musicians and musicians with factors regularity, cue and anterior-posterior distribution indicating an effect of cue (time window 300 – 350 ms: $F(1,19) = 4.38$, $p = 0.05$, partial $\eta^2 = 0.19$) and an interaction between regularity, cue, and anterior-posterior distribution (time window 300 – 350 ms: $F(1,19) = 7.75$, $p < 0.05$, partial $\eta^2 = 0.29$; time window 400 – 450 ms:

$F(1,19) = 4.46$, $p < 0.05$, partial $\eta^2 = 0.19$) only in musicians. Follow-up ANOVAs conducted separately for anterior and posterior ROIs with factors regularity and cue in musicians, indicating an effect of cue (time window 300 – 350 ms: $F(1,19) = 5.33$, $p < 0.05$, partial $\eta^2 = 0.22$; time window 400 – 450 ms: $F(1,19) = 3.07$, $p < 0.05$, partial $\eta^2 = 0.14$) only in anterior ROI. Paired-sample *t*-tests comparing ERPs between cued and non-cued conditions for the anterior ROI in musicians, reflected that ERPs elicited in the cued condition were larger than in the non-cued condition for the anterior ROI (time window 300 – 350 ms: $t = 2.31$, $p < 0.05$, Cohen's $d = 0.38$; time window 400 – 450 ms: $t = 2.09$, $p < 0.05$, Cohen's $d = 0.33$; see gray-shaded areas in Fig. S1).

Because effects were not observed in multiple successive time windows, these results have to be treated with caution. Therefore, we provide the data and analysis of the penultimate chords here to motivate hypothesis of future studies, but we will not discuss them further.
